# MOMENTARY ASSESSMENT OF SOCIAL DISCONNECTION IN OLDER ADULTS AT HEIGHTENED RISK FOR SUICIDE

**DOI:** 10.1093/geroni/igae098.1879

**Published:** 2024-12-31

**Authors:** Emily Bower, Taylor Loskot, Kimberly Van Orden

**Affiliations:** Pacific University, Hillsboro, Oregon, United States; Pacific University, Hillsboro, Oregon, United States; University of Rochester Medical Center, Rochester, New York, United States

## Abstract

Social disconnection is a proximal and modifiable risk factor for suicide among older adults. Identifying periods of acute or intensified disconnection can inform just-in-time interventions for individuals at heightened risk for suicide. Ecological momentary assessment (EMA) using mobile devices is feasible and acceptable for older adults, and is a promising method for capturing daily changes in social disconnection. To assess preliminary validity of EMA for assessing social disconnection in older adults at heightened risk for suicide, we compare mobile EMA to paper-and-pencil assessments of social disconnection within a sample of older adults with subjective cognitive decline (n=27, mean age=69 years, 81% female). Participants completed baseline paper-and-pencil measures of social disconnection (INQ) and depression symptoms, then completed daily surveys (EMA) that were delivered to their smartphone up to 4 times a day for 14 days. Three EMA LIkert-type items items assessed aspects of social disconnection (burdensomeness, belonging, and disconnection). Participants completed a mean of 37.2 surveys with an average response rate of 67%. EMA items correlated strongly with INQ scores (r’s=.6-.7) and depression (r’s=.5-.7). Intercorrelations among EMA items were low to moderate (r’s=.3-.6), suggesting items assessed unique aspects of social disconnection. Among 19 participants who reported no perceived burden on the INQ, 63% reported daily variability in the EMA burden item. Additional analyses will examine within-person variations in EMA items using multilevel mixed models. Findings provide initial evidence of validity for mobile EMA to assess momentary changes in social disconnection among older adults at heightened risk for suicide.

